# Infections of horses and shrews with Bornaviruses in Upper Austria: a novel endemic area of Borna disease

**DOI:** 10.1038/emi.2017.36

**Published:** 2017-06-21

**Authors:** Herbert Weissenböck, Zoltán Bagó, Jolanta Kolodziejek, Barbara Hager, Günter Palmetzhofer, Ralf Dürrwald, Norbert Nowotny

**Affiliations:** 1 Institute of Pathology and Forensic Veterinary Medicine, University of Veterinary Medicine Vienna, Vienna A-1210, Austria; 2 Institute for Veterinary Disease Control Mödling, Austrian Agency for Health and Food Safety (AGES), Mödling A-2340, Austria; 3 Institute of Virology, University of Veterinary Medicine Vienna, Vienna A-1210, Austria; 4 Veterinary Practice St. Agatha, St. Agatha A-4084, Austria; 5 Veterinary Practice Hartkirchen, Hartkirchen 4081, Austria; 6 Department of Basic Medical Sciences, College of Medicine, Mohammed Bin Rashid University of Medicine and Health Sciences, Dubai Healthcare City, Dubai 505055, United Arab Emirates

**Keywords:** Austria, bicolored white-toothed shrew, Borna disease virus-1, common shrew, *Crocidura leucodon*, endemic area, horse, reservoir

## Abstract

Borna disease, a lethal infection with Borna disease virus-1 (BoDV-1), was diagnosed in four horses from Upper Austria in 2015 and 2016. All cases occurred in winter (two cases in February 2015 and two cases in December 2016), and the maximal distance of the affected stables was 17 km. To demonstrate whether the causative agent was also harbored by its reservoir host, the bicolored white-toothed shrew (*Crocidura leucodon*), 28 shrews from this geographic area were collected in 2015 and investigated for the presence of BoDV-1. The shrew species were identified according to taxonomic clues and molecular barcodes. Affected horses and all shrews were investigated using histology, immunohistochemistry (IHC) and reverse transcription PCR. The horses exhibited severe nonpurulent encephalitis. Large amounts of BoDV-1 antigen were identified in their CNS. Among the 28 shrews, nine were identified as *C. leucodon* and 13 as *Sorex araneus* (Common shrew; Eurasian shrew). Six *C. leucodon* (66.7%) and one *S. araneus* (7.7%) had BoDV-1 infections. In accordance with previous findings, the IHC of *C. leucodon* exhibited a high amount of viral antigen in many neural and extraneural tissues. By contrast, the single positive *S. araneus* had an exclusively neural staining pattern. Of all positive samples, whole-genome BoDV-1 sequences were generated. The acquired sequences of the affected shrews were not identical to each other and clustered around the sequences of the diseased horses belonging, surprisingly, to the German ‘strain V’ cluster.

## INTRODUCTION

Borna disease virus-1 (BoDV-1) is the causative agent of Borna disease (BD), a neurological condition of horses and other mammals, which has been credibly identified only in certain endemic areas in central Europe to date.^1, 2, 3^ The affected regions include eastern and southern Germany, the eastern part of Switzerland and the area bordering Liechtenstein, and the most western federal state of Austria, Vorarlberg.^2^ In an area not associated with these endemic areas, in the Austrian federal state of Styria, a single case of a lethal infection with a divergent bornavirus termed Borna disease virus-2 (BoDV-2) was detected ~20 years ago.^4^ Increasing evidence suggests that the natural reservoir host of BoDV-1 is the bicolored white-toothed shrew, *Crocidura leucodon*, and that disease in large mammals is only an accidental spillover event.^5, 6, 7^ In *C. leucodon*, the virus replicates in a large number of tissues and is excreted by different routes, but pathological lesions and clinical signs are not reported.^8^ In large mammals, such as horses, the infection is selectively neurotropic and triggers a massive infiltration of T cells into the central nervous system (CNS), ultimately leading to nonpurulent encephalitis, characteristic clinical signs and an almost inevitable lethal outcome.^9^

Antibodies to Bornaviruses were also identified in different species outside of the endemic areas in Germany and other countries.^10, 11, 12^ Owing to the broad cross-reactivity between mammalian and avian Bornaviruses, antibody findings cannot be considered proof of infection with mammalian Bornaviruses.^13^

Despite the claims of several publications of the presence of BoDV-1 in regions of the globe remote from the classical endemic areas, these findings have not been confirmed epidemiologically, and the recovered virus sequences are compatible with sample contaminations with common laboratory strains.^14, 15, 16^

The recent identification of a divergent bornavirus in squirrels (Variegated squirrel*; Sciurus variegatoides* and Prévostś squirrel; *Callosciurus prévosti*) and the association of this virus with deaths in humans has increased the attention of the scientific community on this viral family.^17, 18^

Thus, in the context of this changing landscape of bornavirus research, the purpose of this study is to present evidence for a new endemic area of BoDV-1 documented by both clinically affected horses and the presence of the virus in two different shrew species.

## MATERIALS AND METHODS

**Figure 1:**
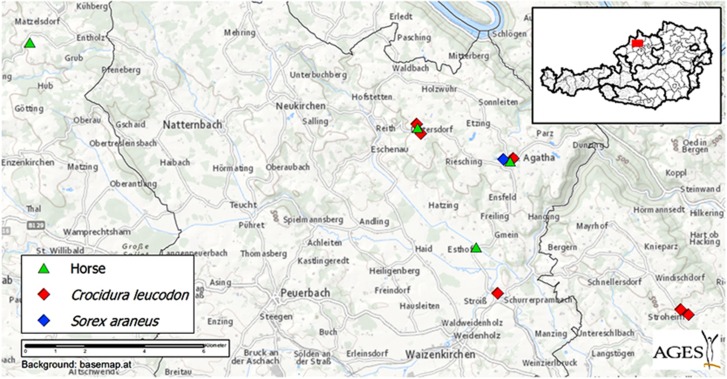
Map depicting the geographical distribution of the BoDV-1-positive horses and shrews. The inset shows the area in Upper Austria, which has been enlarged.

In February 2015 and December 2016, a total of four horses, all originating from different small family-owned premises in Upper Austria, were humanely killed due to progressive neurological signs (Figure 1). The most common clinical signs included depression, ‘pipe smoking’ behavior, and ataxia (Table 1).

The head and selected organ samples of the affected animals were sent to the national reference laboratory for equine encephalomyelitis to exclude notifiable infectious diseases. After removing the brain under strict biosafety measures, standard histopathological and immunohistochemical investigations and molecular analyses for the exclusion of rabies, flaviviruses and American encephalomyelitides were performed. On the basis of histopathological findings, subsequent analyses for BoDV were carried out.

To investigate the existence of a local virus reservoir, 28 shrews were collected between April and October 2015 in two districts of Upper Austria in close proximity to the properties with 3 of the affected horses (Figure 1). These shrews were either caught by cats or trapped inadvertently in commercially available rodent traps. The animals were kept frozen at −20 °C until transportation to the laboratory, where necropsy was performed immediately after complete thawing. The shrew species were identified according to taxonomic clues^19^ and molecular barcodes. Molecular confirmation of all shrew species was performed by amplification and sequencing of a fragment within the mitochondrial DNA cytochrome b (mtDNA cytb) gene. For this purpose, published PCR assays were performed using two universal primer pairs: L14724f with H15915r^20^ and/or L14724f (as before) with H15149r.^21^ For the genomic DNA-PCRs, a Fast Cycling PCR Kit (Qiagen, Redwood, CA, USA) was applied. At necropsy, tissue samples from the brain and skin of all shrews and from the lung, heart, liver, spleen, kidney and intestinal tract of 24 animals were fixed in 10% neutral buffered formalin. Brain samples from all shrews were immediately frozen at −80 °C.

The formalin-fixed horse and shrew samples were processed to paraffin blocks, sectioned at 2 μM and placed on Superfrost plus slides (Thermo Fisher Scientific, Fremont, CA, USA). The slides were stained with hematoxylin and eosin and subjected to immunohistochemistry with the anti BoDV-antibody Bo18. This antibody recognizes a peptide (LYEPPASLP) in the nucleoprotein of the vast majority of known strains of BoDV-1 and BoDV-2.^22^ The staining procedure was performed with an automated immunostainer (Thermo Autostainer 360-2D System, Thermo Fisher) using the Ultravision LP Detection System (Thermo Fisher). The dilution of the primary antibody was 1:30 000.

Four different brain regions (brain stem, cerebellum, hippocampus and cerebral cortex) and the trigeminal ganglia of all horses were investigated by BoDV reverse transcription PCR (RT-PCR). In addition, the spleen of the first horse, cerebrospinal fluid and lymph nodes of the second horse, and basal ganglia and mesencephalon of the third horse were included. All frozen samples of the horses and the frozen brain samples of 28 shrews were thawed quickly and homogenized by addition of nuclease-free water (ROTH, Karlsruhe, Germany) and ceramic beads (VWR International, Vienna, Austria) using an automatic TissueLyser II (Qiagen). The obtained tissue suspensions were centrifuged at 10 000*g* for 5 min, and the supernatants were processed by automated nucleic acid extraction (QIAcube, Qiagen) using a QIAamp Viral RNA Mini QIAcube Kit (Qiagen) according to the manufacturer’s instructions. This method allows the use of the same extracts to detect both viral RNA and genomic DNA.

All BoDV RT-PCR assays were performed with the OneStep RT-PCR Kit (Qiagen). The nucleic acid extracts were first investigated with the published screening BoDV RT-PCR.^23^ All BoDV-positive samples were subsequently identified by 6 further RT-PCRs targeting the complete N, X and P genes and the N/X intergenic region. To each reaction mix containing PCR buffer, dNTPs and enzymes (amounts according to the manufacturer’s instructions), 0.4 μM of the corresponding primer pair, and 10% template were added. Reverse transcription and denaturation steps were determined according to the manufacturer’s instructions. The DNA amplification was performed in 45 cycles of heat denaturation at 94 °C for 30 s, primer annealing at 60 °C for 30 s and DNA extension at 72 °C for 30 s followed by a final extension at 72 °C for 7 min. Given that all of the horse samples were positive by the screening PCR, only the cerebrum samples were chosen for the subsequent detailed investigations. The specific amplification products were purified using PCR Kleen Spin Columns (Bio-Rad, Hercules, USA) following the manufacturer’s protocol and subjected to sequencing by Microsynth (www.microsynth.ch) or by Eurofins (www.eurofinsgenomics.com). Each PCR product was sequenced in both directions.

As the first sequence analysis exhibited the highest identity to BoDV-1 strain V (GenBank acc. no U04608), the subsequent primer pairs for the completion of the whole BoDV-1 genomes were designed based on this nucleotide sequence. A total of 13 new primer pairs (targeting the complete M, G, and L genes) were created using the Primer Designer program (Scientific and Educational Software, Durham, NC, USA) and synthesized by Microsynth. The primer sequences are listed in Table 2. The newly established RT-PCR assays were conducted for each positive sample as described above, and the PCR products were sequenced.

All obtained BoDV-1 sequences were manually verified and compiled to continuous full genome sequences. The sequences were then compared to each other and to the bornavirus sequences from GenBank. Prior to the phylogenetic analysis, ClustalW multiple sequence alignments were conducted using BioEdit Sequence Alignment Editor Version 7.0.9.0. Several phylogenetic trees were created with the MEGA7 program^24^ using both the neighbor-joining (NJ) and the maximum-likelihood (ML) methods with different algorithms by employing 1000 replicates of bootstrap resampling analysis for each tree. Two trees with the highest supporting bootstrap values were chosen.

The first phylogenetic tree was inferred on the basis of complete coding sequences of 15 representative members of the genus *Bornavirus* and eleven sequences determined in this study. The second phylogenetic tree including only selected BoDVs-1 was constructed from 54 1824-bp long sequences coding for the N gene, the N/X intergenic region, and X and P genes.

For investigation of potential genome integration of BoDV, one primer pair within the N gene^23^ and another within the P gene^2^ were used, and PCRs without the RT-step were performed using a Fast Cycling PCR Kit.

For detection of the new agent Variegated squirrel bornavirus-1 all samples were tested using the recently described BoDV-like RT-qPCR (assay 6).^17^

## RESULTS

**Figure 2:**
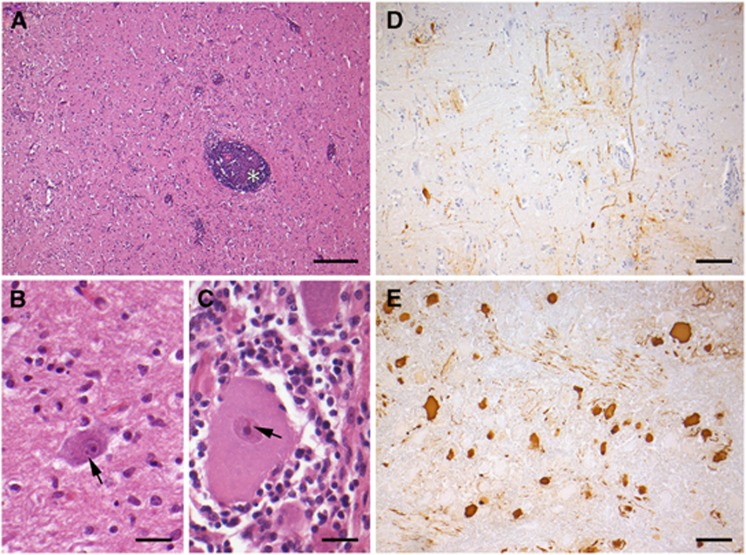
Bornavirus encephalitis in horses. (**A**) Severe nonpurulent encephalitis with perivascular cuffing (asterisk) and microgliosis. Bar=250 μm. (**B** and **C**) Eosinophilic intranuclear inclusion bodies (arrows) in a brainstem neuron (**B**) and a trigeminal ganglion neuron surrounded by lymphohistiocytic infiltration (**C**). Bars=25 μm. (**D** and **E**) Immunohistochemical demonstration of BoDV antigen (brown signals) in neurons and glial cells and their processes. Bars=100 μm. (**A**–**C**) Hematoxylin and Eosin staining. (**D** and **E**) Bo-18 immunohistochemistry.

All horses were negative for notifiable infectious viral encephalitides, such as rabies, flavivirus and alphavirus encephalitides. Histologically, all affected horses exhibited moderate to severe nonpurulent encephalitis with perivascular lymphohistiocytic cuffing, microgliosis, neuronal necrosis and eosinophilic intranuclear inclusion bodies (Joest Degen) in neurons. In addition, nonpurulent inflammation of trigeminal ganglia (in horse no. 2 with demonstration of Joest Degen inclusion bodies in ganglion cells) was consistently identified. Based on immunohistochemistry, high amounts of BoDV antigen were present in neurons, glial and ganglion cells and their processes (Figure 2; Table 1).

Among the 28 shrews, 9 were identified as *C. leucodon,* 13 as *Sorex araneus* (Common shrew, Eurasian shrew), 3 as *Neomys anomalus* (Miller’s water shrew), 2 as *C. suaveolens* (Lesser white-toothed shrew) and one as *S. minutus* (Pygmy shrew).

**Figure 3:**
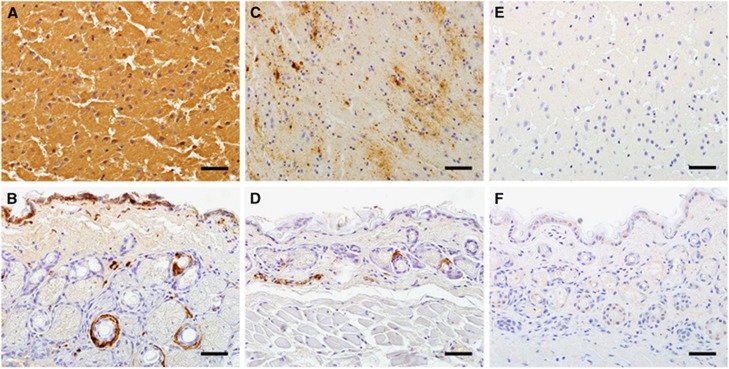
Bornavirus infection of shrews. (**A** and **B**) Demonstration of BoDV-1 antigen in an infected *Crocidura leucodon* shrew. In the brain (**A**), the antigen is diffusely distributed; in skin (**B**), keratinocytes and periadnexal nerve fibers are positive. (**C** and **D**) Demonstration of BoDV-1 antigen in an infected *Sorex araneus* shrew. In the brain (**C**), the viral antigen has a multifocal, patchy staining pattern, representing individually stained neurons and glial cells and their processes. In skin (**D**), the viral antigen is confined to periadnexal nerve fibers; keratinocytes are negative. (**E** and **F**) Lack of immunoreactivity in brain (**E**) and skin (**F**) in a non-infected *C. leucodon* shrew. Bars=40 μm; Bo-18 immunohistochemistry.

Six *C. leucodon* (66.7%) and one *S. araneus* (7.7%) were clearly positive for BoDV infections by both IHC and RT-PCR. All *C. leucodon* exhibited a high amount of viral antigen in neural tissues, such as brain, peripheral and vegetative nerve fibers, and in a number of extraneural tissues, such as epidermis, salivary glands, bronchiolar epithelium, smooth muscle cells, myocardium and adipose tissue. The parenchyma of large organs, such as liver, kidney, lung and spleen, exhibited no specific staining signals. Although staining of neural tissue was consistent, diffuse and strong in all animals, the staining pattern in other tissues was variable and not present in each individual. The single positive *S. araneus* exhibited a different staining pattern. In the brain, multifocal staining of groups of neurons and glial cells was noted, where the signals could be clearly attributed to nuclei, perikarya or processes of individual cells. The only other investigated tissue in this case was the skin, where the keratinocytes were negative but periadnexal and dermal nerve fibers exhibited positive signals (Figure 3). All shrews including the BoDV-1-positive individual of *S. araneus* displayed no inflammatory infiltrations in the brain and other organs.

From the brain samples of four horses and seven shrews, whole-genome BoDV sequences were generated. The sequences exhibited 99.6% to 99.9% identity to each other. The smallest divergence was observed between viruses identified in horse no. 1 and in two shrews: CL311/15 and CL690/15 (only 3 nts difference in 8 769 coding nts). The highest divergence was observed between shrews CL1285/15, CL1287/15 and horse no. 4 (30 nts). Sequence identities to the German reference BoDV-1 strains V, H1766, and He/80 were 97.8%–97.9%, 97.5%–97.6% and 94.7%–94.8%, respectively. The sequence identity to the Austrian BoDV-2 strain No/98 was only 81.0%–81.1%.

The analysis of 1824-nt long sequences comprising complete N, P, and X genes of 54 BoDVs-1 revealed the highest identity to the German horse isolate H215/FR (between 98.5% and 98.7%) and to further strains and viruses around strain V (between 97.6% and 98.5%).

Furthermore, in one horse sample (no. 2), a P gene fragment was identified by the BoDV DNA-PCR. Its sequence was 100% identical to that of the viral sequence achieved by the BoDV RT-PCR. In addition, P or N gene fragments were integrated into the genomes of two shrews (CL428/15 and CL690/15). The N gene sequence of the genomic BoDV of shrew CL690/15 was 100% identical to its viral sequence, the genomic- and viral-nucleotide P gene sequences of shrew CL428/15 exhibited one nucleotide substitution associated with an amino acid exchange.

Multiple alignments of the corresponding complete proteins between the new strains demonstrated only one (of 370) amino acid substitution within the N gene (in CL428/15), 1 (of 201) within the P gene (in CL1287/15), 6 (of 503) substitutions within the G gene (in 9 different sequences), and 12 (of 1711) within the L gene (in all sequences). Amino acid sequences of the X gene (87 aa) and M gene (142 aa) exhibited 100% identity to each other.

All samples were negative by the BoDV-like RT-qPCR specific for variegated squirrel bornavirus-1, which belongs to the species *Mammalian 2 bornavirus*.

**Figure 4:**
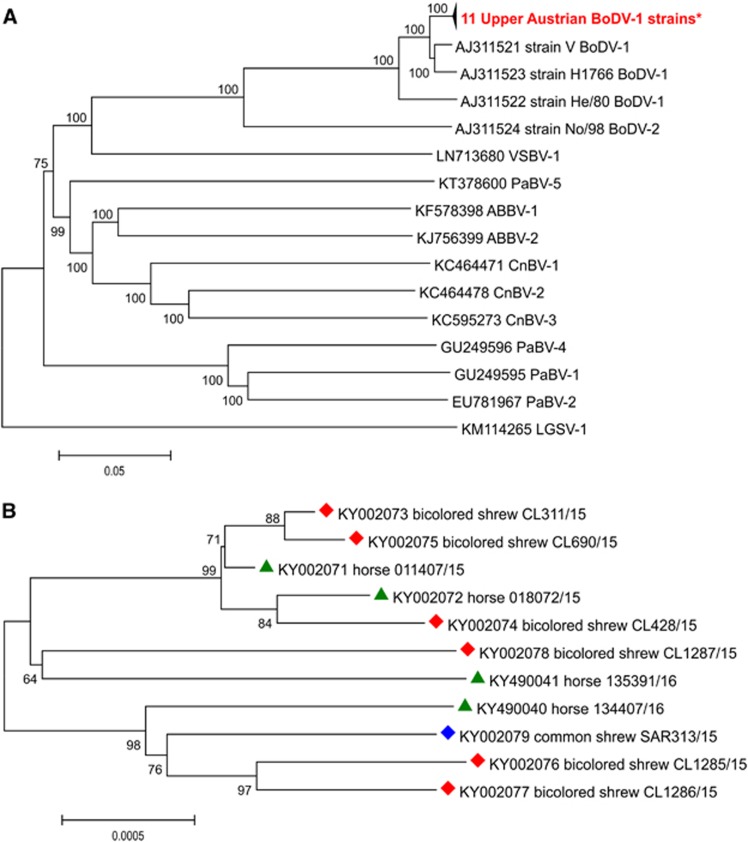
Phylogenetic tree of the complete coding sequences of representative members of the genus *Bornavirus* including new Upper Austrian sequences. (**A**) For better visualization, the eleven sequences determined in this study are collapsed. (**B**) Only the sequences of the new Upper Austrian Bornaviruses are presented. These sequences are marked with green triangles (horse-derived BoDVs-1), red diamonds (*C. leucodon*-derived BoDVs-1), and blue diamond (*S. araneus*-derived BoDVs-1). GenBank accession numbers, strain names (in the case of BoDVs) and species abbreviations^3^ are indicated at the branches. All supporting bootstrap values are displayed next to the nodes. The horizontal scale bar indicates genetic distances. *GenBank acc. nos: KY002971-79 and KY490040-41.

With the current data sets, the NJ method with the p-distance algorithm of MEGA7 was the best method for phylogenetic analysis. Analysis of the full-length recovered genome sequences demonstrated that the new Austrian viruses represent further members of BoDV-1 belonging to the species *Mammalian 1 bornavirus* of the genus *Bornavirus* (Figure 4). These sequences are clearly segregated from the Austrian reference strain No/98, representing the only member of BoDV-2 (Figure 4).

**Figure 5:**
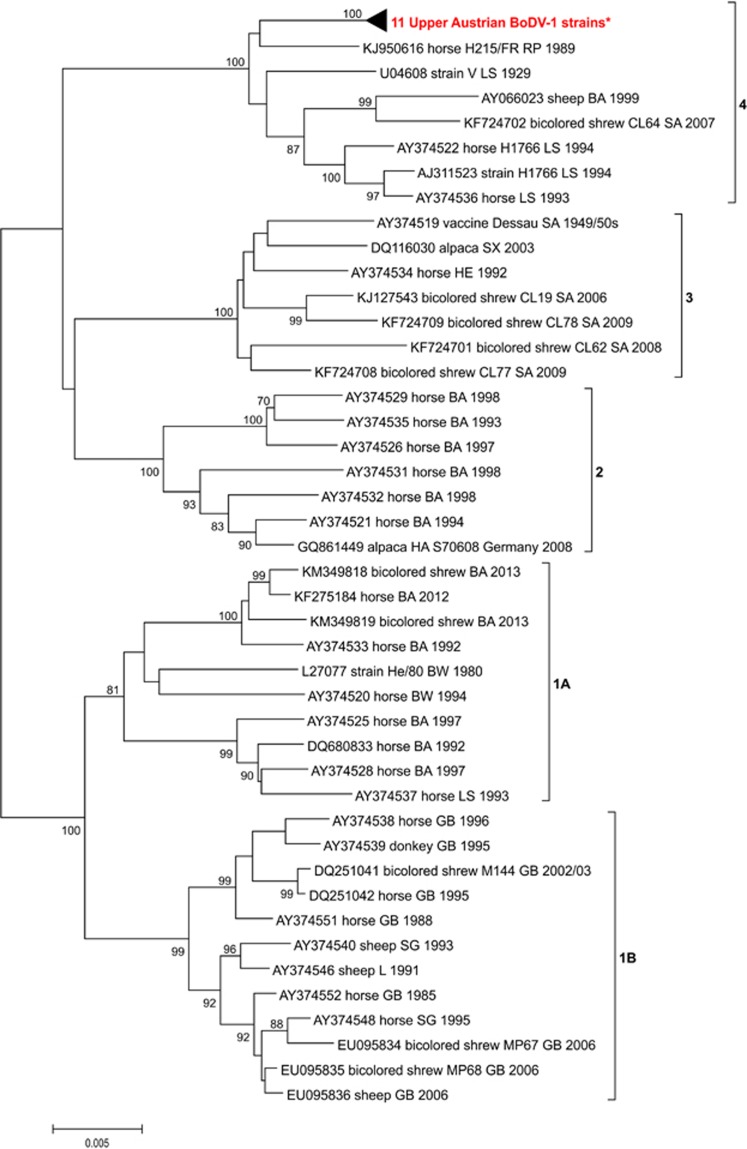
Phylogenetic tree of an 1824-bp long sequence stretch (coding for N protein, intergenic N/X, as well as for X and P proteins) of 43 selected BoDVs-1 from the GenBank and eleven sequences determined in this study (which are collapsed). The five major clusters are indicated. The GenBank accession numbers, host or strain names, geographic location and years of isolations are indicated at the branches. Supporting bootstrap values ≥70% are displayed next to the nodes. The horizontal scale bar indicates genetic distances (here 0.5% nucleotide sequence divergence). BA, Bavaria; BW, Baden-Wurttemberg; GB, Graubuenden; HE, Hesse; L, Liechtenstein; RP, Rhineland-Palatinate; SA, Saxony-Anhalt; SG, Sankt Gallen; SX, Saxony; TH, Thuringia. *GenBank acc. nos: KY002971-79 and KY490040-41.

More detailed phylogenetic investigation of the fifty-four 1824-bp long sequences of only BoDVs-1 exhibits the presence of five different, geographically confined clusters well-known from previous studies (Figure 5).^2, 7^ The first cluster (1A) contains mostly viruses from Southwest Germany closely related to the strains from Switzerland and Liechtenstein (cluster 1B). Cluster 2 represents the South German group and cluster 3 includes Bornaviruses mostly from Southern Saxony-Anhalt and Saxony in Germany. The newly discovered Upper Austrian strains share cluster 4 together with other Central German sequences (Figure 5). These strains cluster close to each other independent of their hosts and collection time. Further, they exhibit close relationships to other German strains and viruses around the oldest BoDV strain V isolated in 1929 in Lower Saxony, especially to strain H215FR, discovered in a horse with clinical BD reported to originate from BoDV-1-non-endemic Rhineland-Palatinate in 1989 and analyzed 25 years later (Figure 5).^25^

The eleven newly described Upper Austrian complete genome BoDV-1 sequences are available from GenBank under the following accession numbers: KY002071 (Horse 011407/15), KY002072 (Horse 018072/15), KY002073 (CL311/15), KY002074 (CL428/15), KY002075 (CL690/15), KY002076 (CL1285/15), KY002077 (CL1286/15), KY002078 (CL1287/15), KY002079 (SAR313/15), KY490040 (Horse 134407/16) and KY490041 (Horse 135391/16).

## DISCUSSION

This study clearly demonstrates that the map of endemic areas for BoDV-1 is far from complete and that new endemic pockets remote from the well-known affected regions may emerge from time to time. In the present scenario, confirmed BD cases occurred within a short time span in four horses housed in stables not much more than 17 km apart. Another horse with matching clinical signs and seropositive for BoDV-1 was already registered in the same area in late 2014. This case was not subjected to laboratory investigation post mortem; thus, a definitive diagnosis has not been made (personal observation, unpublished). The closest holdings of equines diagnosed with BD in neighboring Bavaria were at a distance of greater than 70 km.^26^ Intriguingly, phylogenetic analysis revealed that the most closely related BoDV strain was not one of the sequenced strains from Bavaria but was a strain derived from a horse of Rhineland-Palatinate more than 400 km away. The finding of BoDV-1 of cluster 4 (‘strain V group’) in Upper Austria indicates that this group is unique among BoDV-1 because it covers a wide area with a distance greater than 700 km from the north to the south interspersed with regions in which viruses of the other clusters occur. Although BoDV-1 cluster 4 was reported from Bavaria, it has been only found in Franconia to date, a region remote from Upper Austria.^27^

The investigations revealed that some individuals integrate bornavirus-like elements into their genome after infection, which is consistent with findings reported previously.^28, 29^ Signals of members of *Mammalian 2 bornavirus* (variegated squirrel bornavirus-1) were not identified in the shrews and horses, indicating that these viruses establish a completely different infection cycle.

Several shrew species are distributed in Upper Austria, including *C. leucodon*.^30^ The trapping rate of bicolored white-toothed shrews in summer is generally low, indicating a summer habitat outside of farms.^30^ The presence of BoDV-1 in a fairly high percentage of *C. leucodon* shrews, which coincided with overt BD in horses, suggests a high virus burden in farms of this particular region of Upper Austria. The first demonstration of BoDV-1 in a shrew species different from *C. leucodon* can also be interpreted in this context. The different distribution pattern of the virus in the positive *S. araneus* shrew is more consistent with the distribution pattern in accidental hosts, such as horses, sheep and certain zoo animals or experimentally infected adult rats,^9, 31, 32^ and is thus most likely a spillover event due to high viral burden in the environment. However, this could also represent an earlier time-point of infection. In contrast to horses and sheep with overt BD, no inflammation was noted in the brain of this common shrew, indicating a natural resistance against BoDV-1-induced immunopathology.

This is the first report of BoDV-1 infections in Upper Austria. It is unknown when and how the virus became established in this particular region. Given that even in classical endemic areas clinical equine infections occur rarely and unpredictably, overt Borna disease in spillover hosts, such as horses and sheep, is an unreliable indicator of the presence of BoDV-1. In such areas, equine cases may occur at intervals of several decades,^33^ and there are areas with a high viral burden in shrews in which BD has never been recognized.^7^ Thus, the virus could have been present and unnoticed in the region for a long period of time. Recently, favorable events may have caused an increase in the reproductive rate of shrews and driven a larger number of them into horse stables compared with previous years. Thus, the likelihood of equine infections by excretions and sheddings of the shrews into the horse environment increased the risk of infection and ultimately culminated in the lethal spillover infection of the reported four horses. The point in time of overt disease in the four reported horses is more uncommon compared with the seasonal pattern of Borna disease described in Germany, which has a nadir in late autumn and early winter.^14^ Considering the long time period from infection to expression of disease (the incubation period is considered to be up to five months),^7^ the infection may have been the result of previously unknown conditions.

The clinical signs and pathological changes in the four affected horses match the descriptions of classical BD in this species.^34, 35^ Immunohistochemistry revealed a strictly neurotropic infection with clear restriction to the CNS. In this host, the virus did not spread via the peripheral or autonomic nerves, and there were no viral signals in any tissue except the CNS. This tight confinement of the virus to the CNS compartment, which is most likely the result of a strong antiviral cellular immune response, argues against the possibility of viral shedding by infected horses. By contrast, infection in *C. leucodon* is clinically inapparent and seems to persist for the entire life span of the animal. In addition, infectious virus is continuously shed by various routes, such as the saliva, lacrimal fluid, urine, and exfoliated skin.^8^ Contrary to numerous other proven or suspected reservoir hosts for viruses of the order *Mononegavirales*, especially bats and rodents,^36, 37^ the reservoir status of *C. leucodon* has not only been defined virologically but also by demonstration of large amounts of viral proteins and nucleic acids in many organs and tissues. Viral replication in these sites does not seem to negatively influence cell functions and does not lead to any degenerative or inflammatory changes. There is obviously a perfect equilibrium between host and virus that underlines the presumptive natural host status of this shrew species. As previously mentioned,^8^ whether lack of any tissue reaction in BoDV-1-infected *C. leucodon* is due to attenuated pathogenicity, differences in viral entry and circumvention of the antiviral host immune system remains unexplained. The lack of any immunopathological response despite the replication of the virus in a broad range of tissues is compatible with an immunotolerant state, which is reminiscent of experimental rat models of BoDV-1 infection. In certain rat strains, infection in newborns does not trigger an immune response that protects the animals from encephalitis on one hand and that enables the replication of the virus outside the CNS compartment on the other hand.^38^ The same effect is produced by immunosuppressive drugs in adult rats.^31^ Although not fully comparable, intriguing parallels are noted between these experimental models and the naturally infected *C. leucodon* shrews. At present, it can only be hypothesized that the animals are exposed to the virus as newborns by their persistently infected mothers when their immune system is still immature, and that they are immunotolerant to the virus for the remainder of their lives. Given that a few bicolored shrews in Germany also reflected the restriction of BoDV-1 to the brain,^7^ both infection cycles—infections of newborns and spillover to adults—may exist in shrews. Despite the restriction of bornavirus to the brain in these shrews, BoDV-1-positive PCR signals could be detected in the lungs and stomach, reflecting a viral burden in their surroundings and possible mechanisms of virus uptake.^7^ In addition to the finding of BoDV-1-positive shrews in Switzerland and Germany and related spillovers of the infection to horses, this report confirms the role of shrews in the transmission of BoDV-1 in a third country.

## Figures and Tables

**Table 1:** Overview of clinical signs and histopathological findings in the affected horses

**Number**	**Breed**	**Age**	**Sex**	**Clinical signs**	**npE**	**npG**	**JD IB**	**IHC (BoDV)**
1 011407/15	Austrian warm blood	6 y	Mare	Constipation, colic, proprioception disturbances, circular movement, ataxia, disorientation.	+++	+	(+)	+++
2 018072/15	Shetland pony	3 y	Mare	Depression, empty chewing/pipe smoking, proprioception disturbances, circular movement, ataxia, stringhalt.	++	++	++	+++
3 134407/16	Noriker	6 y	Mare	Apathy, depression, pipe smoking, swallowing disorders.	++	+	+++	+++
4 135391/16	Trotter	10 y	Gelding	Initially fever (39.7 °C), loss of appetite, somnolence, ataxia, dyspnea, compulsive walking.	+++	+++	++	+++

Abbreviations: BoDV, Borna disease virus; IHC, immunohistochemistry; JD IB, Joest Degen inclusion bodies; npE, nonpurulent encephalitis; npG, nonpurulent ganglionitis; y, year; (+), single, +, mild, ++, moderate, +++, severe.

**Table 2:** Sequences of 13 primer pairs used in addition to previously described primers^2^ for the generation of complete BoDV-1 genomes

**Primer name/position/direction**	**Primer sequence 5′-3′**	**Length of PCR product (nt)**
BoDV 1566 f BoDV 2372 r	AGA CAT CTC GGC TCG TAT CGGTC GCC TTA TCT CCA GGT CA	807
BoDV 1953 f BoDV 2812 r	CAC ACT GAT GCT TGA GAT AGGTT CAC GAC TTC TGA CTG TA	860
BoDV 2717 f BoDV 3576 r	GTG AGC CAA CAG GAG CTA GACCT GAG CCT GTA TCC GTA GA	860
BoDV 3332 f BoDV 4208 r	ATG TGG TTC GGC AGG TAC TTATT AGG CAG CTT GTC GTG TC	877
BoDV 4073 f BoDV 4955 r	TTG CCT ACC AGC GCA TAG TGAAG GCC GCT GCA TTG TAC TC	883
BoDV 4237 f BoDV 5124 r	GAC ACA GCC AAG AGC AGA TGCTT AGG CAC GAG CAC AGT CA	888
BoDV 5003 f BoDV 5840 r	GAC CGT CAC GAC TTG TGA ATACA GGT ACA CCA CGG AAG AA	838
BoDV 5647 f BoDV 6475 r	GGC CAA GGT GAT AAT CAG ACCGA ATA AGG CCG ACA TAT CC	829
BoDV 6039 f BoDV 6895 r	CCA GGA TGA GTC GCT ATT GACCT TGA CAG CCG TAT TGG AT	857
BoDV 6327 f BoDV 7169 r	GTG GAT TGA GGA AGC GAT AGGAG AAT CGA AGC CAC GTA CT	843
BoDV 6822 f BoDV 7705 r	GAC ATT GCG GTC ACA CCA TCTTG GAC CTG TCG CAG CAT AC	884
BoDV 7350 f BoDV 8221 r	GTT CGT CCT GGC ATG TGA ACCGC ACA GGT CCA TCT CAA GT	872
BoDV 8034 f BoDV 8880 r	GTC ACG CAA TCA ATC ACA GGAAG CAC TGC ACC ACT GAC AT	847

Abbreviations: BoDV, Borna disease virus; f, forward; r, reverse.

Primer positions refer to the sequence of strain V (GenBank acc. number U04608).
